# Localization and quantification of glottal gaps on deep learning segmentation of vocal folds

**DOI:** 10.1038/s41598-023-27980-y

**Published:** 2023-01-17

**Authors:** Mette Pedersen, Christian Frederik Larsen, Bertram Madsen, Martin Eeg

**Affiliations:** 1 Medical Centre, Østergade 18, Copenhagen, Denmark; 2 Copenhagen Business School, Frederiksberg, Denmark; 3 ME-TA, Copenhagen, Denmark

**Keywords:** Computational biology and bioinformatics, Medical research, Mathematics and computing

## Abstract

The entire glottis has mostly been the focus in the tracking of the vocal folds, both manually and automatically. From a treatment point of view, the various regions of the glottis are of specific interest. The aim of the study was to test if it was possible to supplement an existing convolutional neural network (CNN) with post-network calculations for the localization and quantification of posterior glottal gaps during phonation, usable for vocal fold function analysis of e.g. laryngopharyngeal reflux findings. 30 subjects/videos with insufficient closure in the rear glottal area and 20 normal subjects/videos were selected from our database, recorded with a commercial high-speed video setup (HSV with 4000 frames per second), and segmented with an open-source CNN for validating voice function. We made post-network calculations to localize and quantify the 10% and 50% distance lines from the rear part of the glottis. The results showed a significant difference using the algorithm at the 10% line distance between the two groups of *p* < 0.0001 and no difference at 50%. These novel results show that it is possible to use post-network calculations on CNNs for the localization and quantification of posterior glottal gaps.

## Introduction

The tracking of the vocal folds has been performed manually with high-speed video (HSV) for some years, in the form of the segmentation of the glottis. In recent years, tracking has been automated with deep learning; this is an improvement compared to the time-consuming process of a trained expert performing the segmentation manually^1^. The entire area of the glottis has been the focus in the tracking of the glottis, in various forms^2–4^. From a treatment perspective, the specific regions of the moving glottis are of interest e.g. there is controversy surrounding laryngopharyngeal reflux and insufficient closure in the rear part of the vocal folds. To be able to quantify a rear glottal gap, would allow for evidence-based studies to examine this subject. We propose supplementing a proven open-source convolutional neural network (CNN) for tracking the vocal folds with post-network calculations for the localization and quantification of specific glottal gaps during phonation. The current tracking of the vocal folds results in a glottal area waveform, among others, where information on the various regions is lost. We propose a tool for the automatic localization and quantification of glottal gaps in order to evaluate various voice functions and potentially document treatment effects.

The topic is based on our experience in a laryngological clinic with the equipment for HSV from Richard Wolf GmbH. The equipment automatically combined with kymography, electroglottography attachment, and manual calculation of, among others, the distance between the vocal folds in defined areas and places. These possibilities were an improvement and the developer at Richard Wolf did a great job, also in the time-consuming job of getting the CE certification. The combination of measurements is optimal but manual.

On the market for laryngology, stroboscopy is of great value for classification and defining neoplasms, especially malignancy. For function evaluation, HSV is necessary, because the single movements of the vocal folds can be evaluated^5^. A combination of digital videokymography and automatic electroglottography is possible^6,7^.

### Literature review

Tools for tracking the vocal folds with HSV are available, which are mostly used for the classification of laryngeal disorders, such as active contour modeling and support vector machines (SVMs). Yousef et al.^8^ researched the possibility of using a hybrid approach to detect the vocal fold edges during vibration for larynx acquisition for language analysis, utilizing 116,543 frames from a single subject reading a text with unsupervised machine learning. They combined it with active contour modeling to capture the edges of vocal folds on kymograms from HSV. The results showed an accuracy of 97.4% with an error of + / − one pixel. The paper by Eysholdt et al.^9^ paved the way for analyzing HSV in regard to functionality, using a computer simulation on kymograms from HSVs with 2000 frames per second. Unger et al.^10^ aimed to discriminate between T1a carcinoma, and hyperkeratosis by using a support vector machine learning algorithm, with analyses of 15,000 frames of 30 subjects from phonovibrograms based on HSV. Phonovibrography is not a cross-section of the vocal folds as HSV kymography, but it visualizes the opening and closing of the vocal folds in time in a similar fashion. The purpose was to discern objects obstructing the lumen from the glottis, making it possible to detect abnormalities. The result is a classification between precancerous lesions and T1a carcinoma, with a specificity of 100 and a sensitivity of 100, although the authors suggested a large-scale study to verify their findings^10^.

Comparing neural networks is an important aspect for clinical use. For example, Matava et al.^11^ proposed a machine-learning algorithm for classifying vocal cords and tracheal rings in real-time during videolaryngoscopy for airway management and bronchoscopy. The study is interesting due to the real-time aspect. Cho et al.^12^ compared the accuracy of three neural networks, ResNet, Inception, and MobileNetV1, on a dataset of 775 videos; the two best-performing neural networks were additionally trained using transfer learning to further test if this would increase the accuracy. The accuracy was 84% for ResNet, 78% for Inception, and 64% for MobileNetV1. The sensitivity of ResNet improved from 85.1% to 89.2%, and Inception improved from 75.7% to 86.5% with transfer learning. Gomez et al. focused on the image quality of laryngoscopic images from HSV due to low lighting. Their approach to enhancing low-light images using a convolutional neural network was compared against four state-of-the-art low-light enhancement methods and statistically outperformed each on one no-reference and two full-reference image quality metrics. The proposed convolutional neural network was closest to the 3.6 of the original image on the NIQE metric score with 5.0, compared to the four low-light enhancement methods ranging from 10.03 to 10.93. The interesting aspect of this study is the potential for mediating issues with less image quality from HSV recordings for the higher precision of the tracking of the vocal folds^13^.

The tracking of the movements of the vocal folds in various regions of the glottis is of great interest for the objective analysis of large amounts of data. Limitations of the method are e.g. hourglass slits and some spindle-shaped glottal gaps. With deep learning, there is a new possibility for systematic evidence-based randomized controlled trials (RCTs) of phonating vocal folds based on HSV^14^. We have previously, in a Cochrane review, shown that RCTs are rare in our area^15^. HSV provides the exact movement of the vocal folds, and calculations can be made in detail. Studying hoarseness before and after treatment previously did not change the Glottal Analysis Tools (GAT) parameters significantly^16^. Some measures of the vocal folds in GAT are shown, however, to be better than others^17^. More clinical applications with advanced computer solutions are needed, as suggested by Turkmen and Karsligil^18^. The insufficiency of the rear glottal area is, of course, observed in various circumstances. The quality of HSV images of the larynx has to be optimized greatly. It would be valuable, with careful clinical instructions, to ensure optimal HSV usable with CNNs^19^.

The automatic analysis of the parts of the glottis is described in a paper by Wang et al., who used videolaryngoscopy with 29 frames per second (fps) for the automated glottal tracking of vocal fold paralysis. They evaluated the application of the tracking software Automated Glottic Action Tracking by artificial Intelligence (AGATI) for the quantitative assessment of unilateral vocal fold paralysis, with 77% sensitivity and 92% specificity between control and paralysis patients^20^. The paper from Adamian et al. is based on the same software as the previous paper but focuses on predicting unilateral vocal fold paralysis. A maximum opening angle of fewer than 58.65 degrees predicted unilateral vocal fold paralysis with a sensitivity of 85% and a specificity of 85%^21^.

The focus of the paper by Fehling et al. is on the tracking of vocal folds in high-speed videos for the quantitative analysis of the phonating glottis^22^. The paper seeks to determine which neural network is the most precise in the automatic tracking of the vocal folds. Eighteen different convolutional neural networks were trained on 13.000 high-speed video frames and compared to the ground truth for precision, measured with the DICE coefficient. The highest-performing network was a convolutional neural network using long short-term memory U-LSTM^ce-5 (ce- refers to the position of the LSTM in the layers, ce- is both in the contracting and the expanding layer, 5 being the number of U-net levels). Overall, the test data resulted in a mean DICE coefficient of 85% for the glottis, 91% for the right vocal fold, and 90% for the left vocal fold.

### Deep learning model chosen as basis for post-network calculations

We chose the open-source CNN for the tracking of the vocal folds proposed by Fehling et al. to build the proof-of-concept algorithm on top of it. The chosen CNN was used to segment the images from HSV, and we then applied the proposed algorithm to locate and quantify areas of interest on the glottis.

We chose this CNN because it utilizes the same commercial HSV setup from Richard Wolf GmbH, that we have used to record the videos in our database. The CNN is open-source and the source code is publicly available, which allowed us to apply our post-network calculations^22^.

With these post-network calculations, we sought to define one of the main problems seen, namely insufficient closure of some parts. A solution for objective quantification is elaborated to solve the problem, and many kinds of deviances of the glottis can be elaborated with supplemental post-network solutions.

Based on our clinical experience in a laryngology clinic a novel post-network solution of quantitative objective findings for parts of the glottis is proposed.

### The motivation

The novelty of this study was to supplement an existing CNN with post-network calculations for the localization and quantification of regions with glottal gaps during phonation for objective quantitative analysis. To document the feasibility, we conducted a case–control study with 50 subjects/videos. We aimed to divide the automatic tracking of the glottis into smaller regions.

## Materials and methods

### Data collection

The deep learning program was based on HSV with continuous frames (100 during phonation) of each subject/video taken with a commercial setup (Endocam 5562, Wolf GmbH, Knittlingen, Germany), which records a maximum of 2 seconds in 256 × 256 pixels with 2000 or 4000 frames per second (fps); we used 4000 fps in full color, recorded transorally using a 90° rigid scope, on the intonation of /ah/, and with the frequency of spontaneous speech. The subjects/videos were selected from a database of HSV recordings collected between 2007 and 2019 in an otorhinolaryngology clinic in a medical center by the ENT specialist from our team. The subjects/videos were selected arbitrarily from the database: 20 normal subjects/videos (normal closure of the vocal folds) served as a control group and 30 subjects/videos with a rear glottal gap. 100 consecutive frames from each video, were used to test the post-network calculation using the chosen open-source CNN^22^.

**Figure 1:**
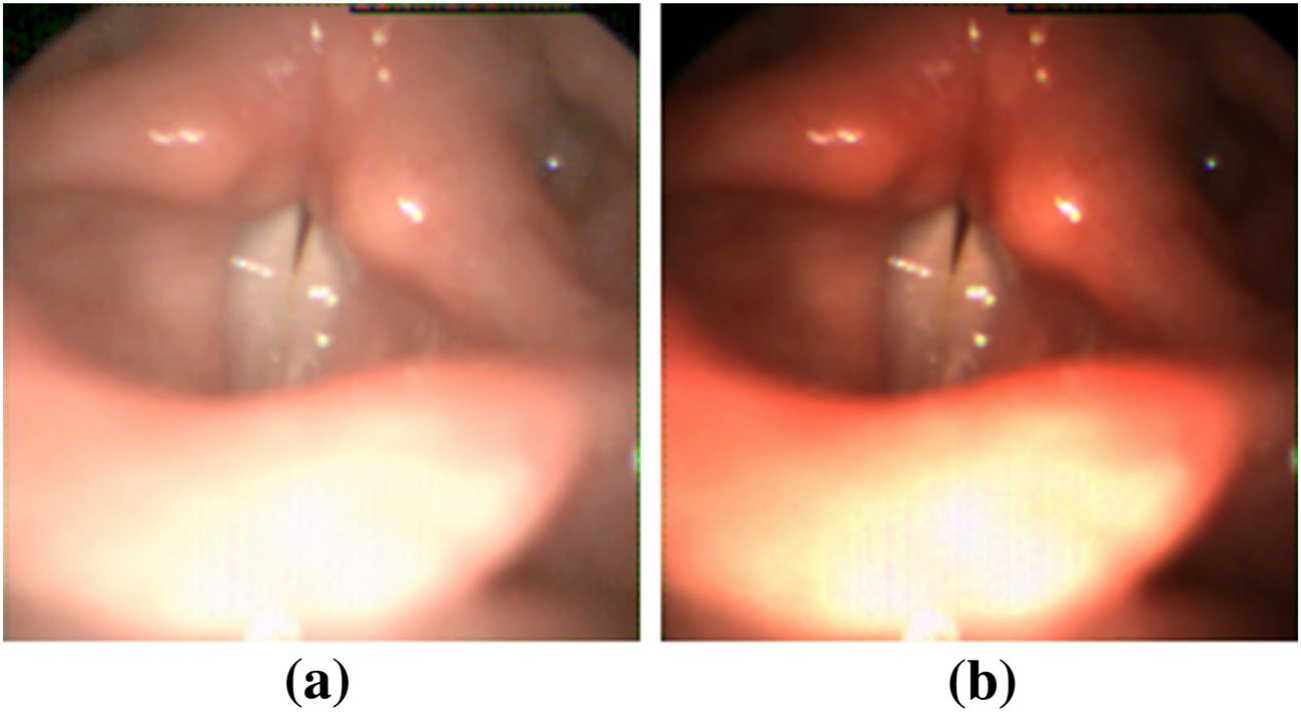
Images from a high-speed video (HSV) recording of one subject with insufficient closure of the rear region of the glottis with and without gamma correction: (**a**) original image; (**b**) gamma-adjusted image with photo-editing software (ImageJ) was used for image processing.

The HSV recordings in our database had a different gamma correction from the one used to train the chosen CNN, resulting in artifacts on several videos. To adjust for this, a gamma correction of + 2 was applied using photo-editing software (ImageJ); Fig. 1 shows an example of an HSV image before and after gamma correction. The images also present an insufficient closure of the rear part of the glottis.

**Figure 2:**
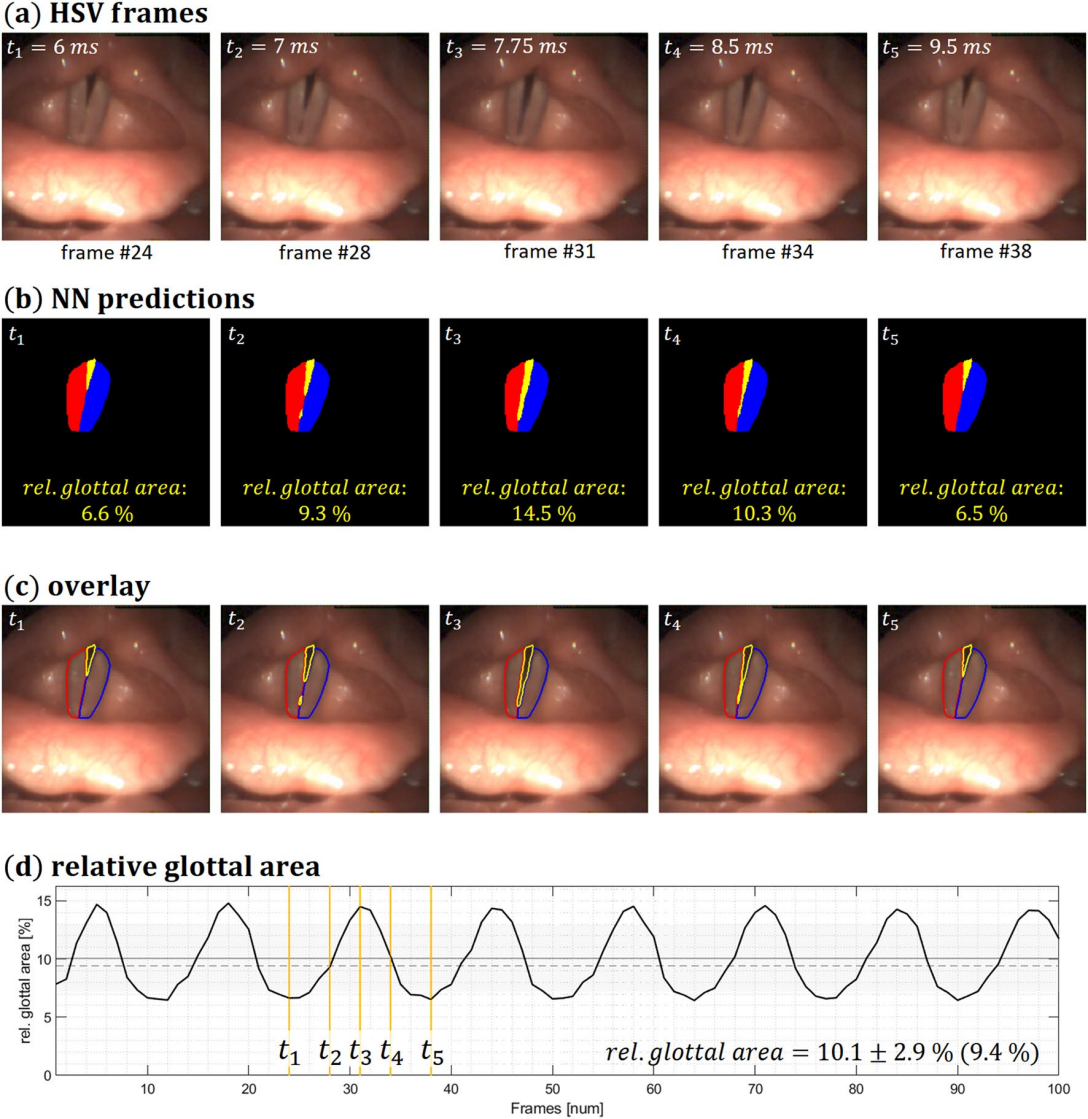
Individual images of the tracking of the vocal folds results and overlays for a single subject with a rear glottal gap: HSV frames; neural network predictions; overlays of predictions on the HSV images; and relative glottal area over time, with yellow markings of the 5 images, made by Fehling et al. using the open-source convolutional neural network, with the U-LSTM^ce-5 architecture, on subjects/videos from our database (reproduced with the approval of the Danish weekly journal for medical doctors ”Ugeskr Læger 2022;184:V02210146″).

Figure 2 shows a comparison of the tracking of the vocal folds, made by Fehling et al., during phonation from 5 out of 100 consecutive frames of the glottal area. The relative glottal area is calculated as the area of the glottis relative to the combined area of the left and right vocal folds and the glottis. This calculation provides no information on which region of the vocal folds a potential insufficiency is located.

**Figure 3:**
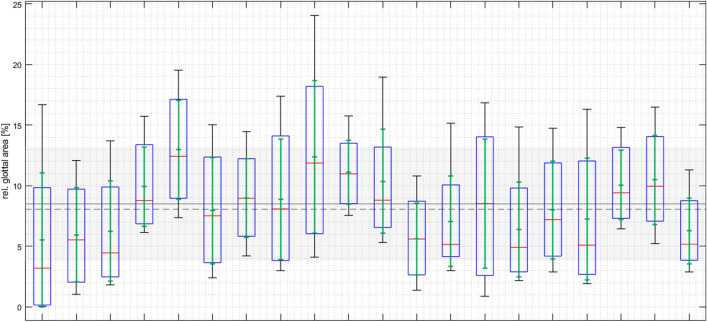
The relative glottal area for sequences with insufficient closure (20 cases): 8.5% ± 4.71% (8.07%). Variations of insufficient closure of the vocal folds during phonation. Twenty subjects/videos with a rear glottal gap included in this paper were chosen from our database and analyzed with deep learning. The highest relative glottal area was 24.04%, and the lowest relative glottal area was 0.05%. The mean highest relative glottal area was 15.7%, and the mean lowest glottal area was 3.5%. None of the 20 subjects with a rear glottal gap had full glottal closure, as shown by the relative glottal area not reaching zero. The relative area is calculated as the glottal area divided by the left and right vocal folds plus the glottal area.

The software can be used to analyze the cycles and detect an insufficient closure of the vocal folds, but not where in the glottis the insufficiency is located. Calculations are presented in Fig. 3, with 20 of the 50 videos included in this paper. The mean highest relative glottal area was 15.7%, and the mean lowest glottal area was 3.5%. The glottal closure was never sufficient, which was documented as the interval of the subjects’ calculations never reaching 0 on the y-axis.

### Post-network calculations

Post-network calculations were performed to optimize the information on specific distances in the glottis. The chosen CNN provides the segmentation but does not provide localized information on the specific distances between the vocal folds. To extract this data we propose an algorithm to serve as post-network calculations. An experiment is presented for calculating the distances between the vocal folds as transverse lines on the glottis at 10% from the rear of the glottis and a comparison at 50% from the rear of the glottis.

The process and steps for post-network calculations were as follows:

After the model is done segmenting the videos, the data are saved as images. The calculations are twofold, the first part is storing the area of the left and right vocal fold along with the area of the glottis and the second part is to calculate the length across the glottis at certain points.

Calculating the area of the three segmented parts is straightforward as the process is simply counting the number of pixels belonging to the different areas, but the process of calculating the length across the glottis is a little more difficult and goes as follows:

First, the image with the biggest opening is found. This is done by using the area of the glottis and selecting the image with the biggest area. This was determined to not only be representative of which image had the biggest opening but also very reliable, as small errors and fluctuations in the segmentation had no major effect on this.

**Figure 4:**
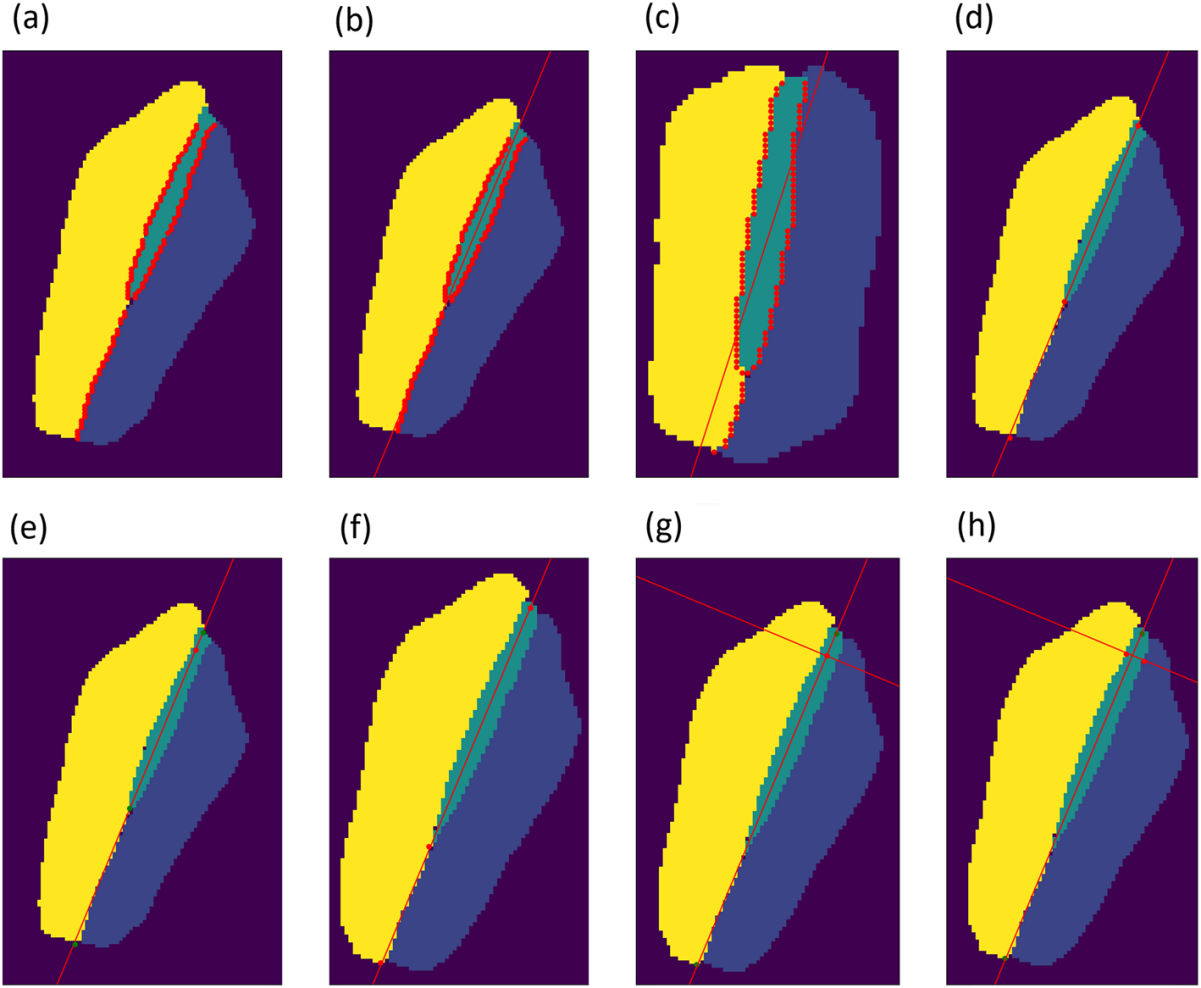
Image representations of the process of localizing a specific line on the glottis for quantification. (**a**) The image with the biggest opening, with points on the edge between the left and right vocal fold. (**b**) linear regression between the points on the edges, using an x over y regression. (**c**) Image of another patient using a y over x regression line. (**d**) Collision points for upper and lower borders are marked. (**e**) The length between the collision points is used to determine the 10% point from the rear of the glottis. (**f**) The algorithm loops through all segmented images and performs the same steps. (**g**) A perpendicular line is drawn at the 10% point from the rear of the glottis. (**h**) Points at the vocal fold edges are placed on the perpendicular line to determine the distance between them at the specific point on the glottis.

**Figure 5:**
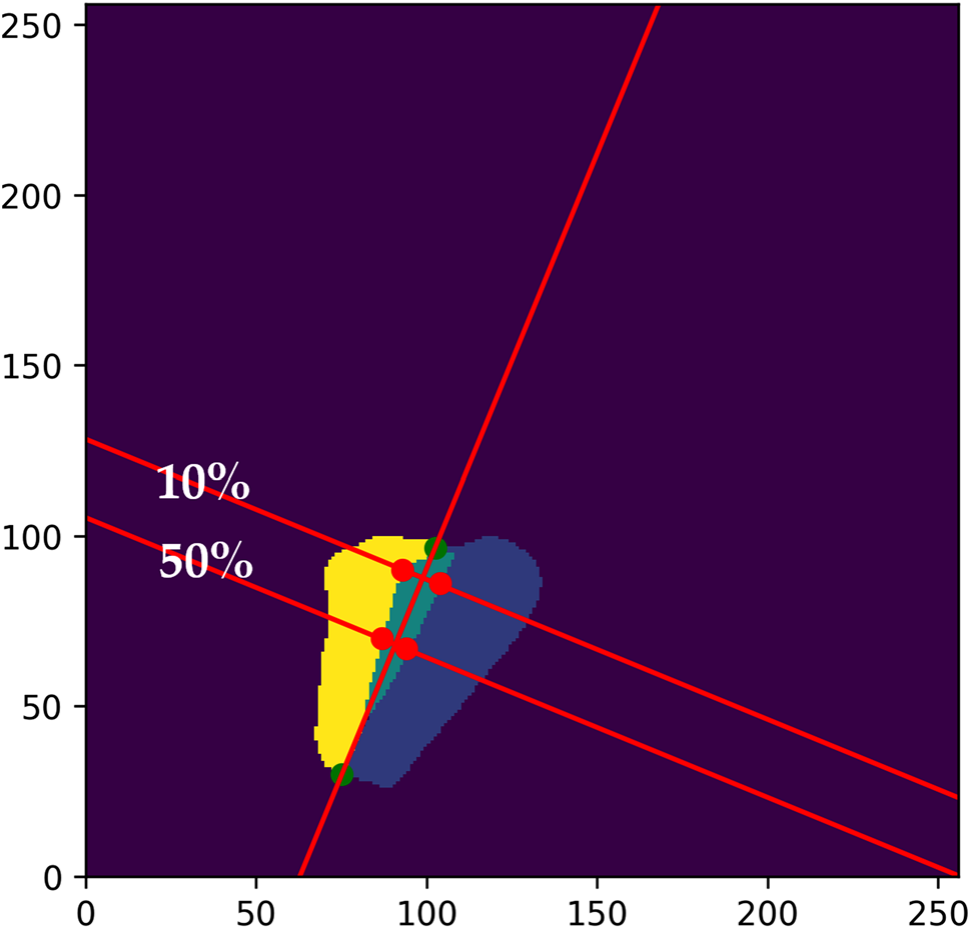
A single image visualizing the post-network calculations of the 10% and 50% lines from the rear of the glottis. The right and left vocal folds are marked in yellow and blue, respectively, and the glottis is marked in teal.

The images were placed in a 256 × 256 coordinate system, with the lower left corner representing (0, 0) as seen in Fig. 5. Points were drawn on the edges between the left and right vocal fold as seen in Fig. 4a. A line between the points is drawn, using the least squares method as seen in Fig. 4b. Instead of doing a y over x regression, an x over y regression is used. This means that, instead of minimizing the vertical distance between the points and the line, the regression is instead trying to minimize the horizontal distance between the points and the line using this formula for the slope:

$$\left( {\frac{{\sum {\left( {x - \overline{x}} \right)} \cdot \left( {y - \overline{y}} \right)}}{{\sum {\left( {y - \overline{y}} \right)} }}} \right)^{ - 1}$$

This is usually not something that makes sense from a purely mathematical perspective, but when done with a visual purpose, where points in the two-dimensional space are not representing a dependent and independent variable, but rather a purely visual indicator of a collision, it suddenly makes sense.

The reason for this is, as the orientation of the vocal fold almost reaches vertical, the more inaccurate a y over x regression becomes at drawing a line through the glottis, as optimizing for the least vertical distance between the points and the line does not make much sense if the desired line is nearly vertical. This creates a line that sometimes does not go through the glottis as seen in Fig. 4c.

4 points are determined, which are the upper and lower collision between the top and bottom of the vocal folds and the line along with the upper and lower collision between the top and bottom of the glottis, as seen in Fig. 4d. The upper collision between the top of the vocal folds, and the collision between the top of the glottis is often the same point, which is the reason for there seemingly only being three points.

The distance between the upper and lower collision between the glottis and the line is determined, and points are drawn between these, so that the distance between the new point and the upper collision is 10% of the distance between the upper and lower collision points, and the distance between the new point and the lower collision is 90% of the distance between the upper and lower collision points as seen in Fig. 4e.

The distance between the upper and lower collision between the vocal folds and the line is determined, and the distance between the point we drew, and the lower collision point is measured, and the latter is divided with the first and the result is stored as the 10% distance.

The algorithm will loop through the data of all the segmented images and complete the process for each to determine the length across the glottis similar to Fig. 4f.

A point is drawn between the upper and lower collision of the glottis and the regression line, so the distance between the new point and the lower collision divided by the total distance between the lower and upper collision is the same as the previously stored 10% distance.

We draw a line perpendicular to the regression line at the 10% distance from the rear as seen in Fig. 4g. The collision between the perpendicular line and the edges of the glottis is measured to quantify the distance between the vocal folds at a specific point as seen in Fig. 4h.

This method for measuring the distance at the upper 10 percent of the glottis allows for keeping a fixed point that can move and rotate with the vocal folds and can handle if the camera zooms in or out.

The algorithm was reproduced for a 50% line between the front and the rear glottis. The visualization of both lines in the full coordinate system is seen in Fig. 5. This was used as a proof-of-concept for determining if the algorithm could identify insufficient closure of the vocal folds at specified points.

The storing of the glottis area (including the left and right vocal folds) and the calculation of the lines across the glottis at 10% and 50% distances from the rear point were performed. The distance was counted in pixels in a 256 × 256 coordinate system. The 50% lines were made for comparison; no difference in the distance during phonation was expected between the two groups at 50%.

### Statistical calculations

Data were analyzed in a linear mixed-effect model using REML, including the group (glottal gap/normal) as a fixed effect and the subject as a random effect; N: number of subjects; and n: number of observations. The group differences between the subjects with a rear glottal gap and the normal subjects are presented as least-squares means (LS means) with a corresponding 95% confidence interval.

The mixed linear model was used because it is an extension of simple linear models to allow for both fixed and random effects, particularly used when there is nonindependence of the data. The SAS statistics show a difference between the 10% lines measured in pixels.

### Institutional review board statement

Ethical review and approval were waived for this study due it being a retrospective case–control study; all the methods were carried out in accordance with the relevant guidelines and regulations.

### Informed consent

Patient consent was waived due to the total incognito of the frames; no identification was possible. No human experiments were conducted and no tissue samples were used, as the focus of this paper was to evaluate if a specific technical equipment used for examination is viable for diagnosis and documentation.

## Results

We made a comparison in a case–control study between 30 subjects/videos with a visual rear glottal gap and 20 normal subjects/videos with complete closure, taken from our database, and segmented with U-LSTM^ce-5 on 100 frames from each video in order to calculate the differences between the two groups^22^. The results presented here were based on the supplementary setup with our post-network calculation.

The results are presented in Table 1 with SAS statistics. There was a statistically significant difference between subjects/videos with a rear glottal gap and normal subjects/videos at the vocal fold distance of 10% from the rear of the glottis (*p* < 0.0001). There was no statistically significant difference at the vocal fold distance of 50% from the rear of the glottis (*p* = 0.50).

**Table 1:** Data were analyzed in a linear mixed-effect model using REML, including the group (glottal gap/normal) as a fixed effect and the subject as a random effect; *N* number of subjects; and *n* number of observations. The group differences between the subjects with a rear glottal gap and the normal subjects are presented as least-squares means (LS means) with a corresponding 95% confidence interval. The mixed linear model was used because it is an extension of simple linear models to allow for both fixed and random effects, particularly used when there is nonindependence of the data. The SAS statistics show a difference between the 10% lines measured in pixels.

Parameter		N	n	LS Mean	Standard error	95% Confidence interval	*p* Value
10%	Normal subjects/videos	20	2000	1.66	0.33		
	Subjects/videos with a rear glottal gap	30	3000	4.44	0.27		
	Diff (subjects/videos with a rear glottal gap–normal subjects/videos)			**2.77**	0.42	(1.93—3.62)	< 0.0001
50%	Normal subjects/videos	20	2000	2.83	0.30		
	Subjects/videos with a rear glottal gap	30	3000	2.83	0.24		
	Diff (subject/videos with a rear glottal gap–normal subjects/videos)			**0.26**	0.39	(-0.52—1.04)	0.50

Significant values are in bold.

Our aim was to get objective quantification results of parts of the glottis usable in randomized controlled trials. The novelty and usability of the post-network calculations were documented in the case–control study (Table 1).

## Discussion

### Post-network calculations

We propose that post-network calculations of various distances between the vocal folds should be performed in relation to vocal fold function when using CNNs for the tracking of the glottis during phonation. This study shows that they can be made in Python and implemented in CNNs for the localization and quantification of insufficient closure. They might be used for the objective vocal fold function analysis of various regions of the glottis and vocal folds during phonation, eventually with simpler CNNs.

Our post-network calculation lines of the distance between the vocal folds were made at 10% and 50% from the rear of the glottis, but an area calculation behind the 10% and 50% lines would probably be better. The resolution of the commercial equipment Endocam 5562, is 256 × 256 pixels and makes the calculations limited to very few pixels. WEVOSYS medical technology GmbH–Germany offers a setup with a spatial resolution of 1440 × 1024 pixels. Images with a higher resolution will improve measurements of the distances across the glottis. The 10% line from the rear of the glottis was used to test if post-network calculations were possible; other distances can be used.

The aim was to be capable of selecting specific regions on the glottis with post-network calculations based on a CNN for a better understanding of the phonating larynx with various functionalities. We started by finding the image in each HSV with the biggest insufficient closed phase of the glottis, using linear regression to calculate the distance between the rear and front collision between the glottis and the midline. This method for measuring the distance of any region in the glottis is effective because it allows for keeping a fixed point that can move and rotate with the vocal folds. The next step is to integrate our post-network calculations into software for real-time calculations.

In our study, the commercial equipment by Richard Wolff was used for videos and was the basis for the chosen CNN as well. The commercial HSV equipment has software for the manual measuring of the distances between the vocal folds in the glottis; in the clinic, it was too inaccurate. The chosen CNN was coded to use 100 frames per patient, but for future implementation, more frames should be considered in newer commercial equipment. The mean vibration for males is reported as being 107–132 Hz, and the mean for females is 196–224 Hz during speech^23^. At 200 Hz, 100 frames from an HSV, with 4000 frames per second, only include five full cycles of the vocal fold.

A differentiation between stroboscopy and high-speed video is important, as the framerate of stroboscopy is mostly between 25 and 60 fps, while high-speed video in our case is 4.000 fps^24^. This provides more pixels in the stroboscopic images, resulting in artifacts in the datasets for the neural networks due to the higher frequencies of phonation. Image quality varies, among other aspects, with different amounts of pixels, and gamma correction also affects the model produced by the neural networks. This is important because the accuracy of the model is dependent on using the same image production as was used for training^19^. There were several challenges to be solved. The HSV recordings in our database had a different gamma correction from the one used to train the CNN as presented in Fig. 1, another was the choice by Fehling et al. to measure the area with glottis and the vocal folds as well, which is why the glottis was calculated relative to the total area of glottis plus left and right vocal fold. Fortunately, the CNN U-LSTM^ce-5 is based on HSV equipment from Richard Wolf GmbH, which has a European CE certificate and is the same as ours thereby avoiding many risks.

### Case–control study

An evidence-based case–control study was performed to test the novel post-network calculations. We chose 30 subjects/videos with a clear visual rear glottal gap and compared them with 20 subjects/videos with a normal visual closure from our database. By comparing the two groups, it was statistically possible to confirm the difference with a significance of *p* < 0.0001.

The perspective is to have the distance between the vocal folds quantified automatically in a defined region of the glottis and integrated into software for routine use combined with HSV in the clinic, preferably in real-time.

Deep learning is a major step forward in understanding the phonating larynx because of the automation, quantification, and standardization possibilities for large amounts of information. An interesting area is tissue evaluation before and after treatment as a supplement to describing movement phenomena^25^. Sharme et al. used Optical Coherence Tomography (OCT) during phonation with a framerate of 250 Hz for subsequent analyses^26,27^. Further studies could define the region of interest in the glottis for OCT, which has been done for some time in ophthalmology^28^.

HSV is not a cheap clinical procedure; stroboscopy is cheaper and has been studied extensively^24^. Advancements are being made to standardize a modular setup for HSV that hopefully will make the equipment and software cheaper on a commercial basis for clinical use^29^. There are many aspects of further research possibilities. A tighter collaboration with ophthalmologists is probably the optimal basis for RCTs in the future. An unresolved problem in laryngology is the lack of funding for HSV examinations from the healthcare system, even if hoarseness is a more and more essential complaint.

## Conclusion

A novel method for precise calculations of distance is presented, with a defined line between the vocal folds at specified positions, as grounds for calculating distances between the vocal folds. The method was evaluated in a case–control study by comparing 30 subjects/videos with a clear visual glottal gap in the rear to 20 subjects/videos with normal glottal closure. As a proof-of-concept, two distances were selected at 10% and 50% from the rear glottis in the videos; these distances can be specified for any location of interest. The post-network calculations, based on the segmentation by an open-source CNN with U-LSTM^ce-5 architecture, resulted in a significant difference of *p* < 0.0001 between the two groups at 10% from the rear glottis and no significant difference at 50% from the rear glottis. The method can be applied for the localization and quantification of rear glottal gaps, to allow for future evidence-based studies on the connection between glottal gaps and various pathologies e.g. laryngopharyngeal reflux, and eventually for future clinical use in combination with optical coherence tomography.

## Data Availability

The datasets and code used during the current study are available from the corresponding author on request.
